# Editorial: From pathways to networks: integration of phytohormones and environmental signals

**DOI:** 10.3389/fpls.2025.1667710

**Published:** 2025-08-08

**Authors:** Takuya Yoshida

**Affiliations:** ^1^ Trans-Omics Facility, National Institute for Basic Biology, Okazaki, Japan; ^2^ Basic Biology Program, The Graduate University for Advanced Studies, SOKENDAI, Okazaki, Japan

**Keywords:** phytohormone signaling, cytokinin (CK), auxin, abscisic acid (ABA), phosphatidic acid (PA), brassinosteroid (BR), sulfated peptide, HPLC-MS/MS

Plants possess a sophisticated capacity to perceive and integrate diverse environmental cues, such as light, temperature, and water availability, allowing them to finely control their growth and metabolism (Gupta et al., 2020; Li et al., 2022). These external signals are integrated with endogenous regulators, particularly phytohormones (Li et al., 2022; Waadt et al., 2022). Whilst major components of individual hormone and environmental signaling pathways have been identified through genetic and molecular studies (Vanstraelen and Benkova, 2012; Kim et al., 2024), growing evidence highlights the extensive cross-talk between these systems. This Research Topic aimed to advance our understanding of the phytohormone signaling network—governed by multiple phytohormones and environmental cues—and featured three original research articles and one review article, covering different aspects of phytohormones derived from studies on several species.

Trifunović-Momčilov et al. investigated the hormonal and metabolic changes during spontaneous shoot regeneration in common centaury, *Centaurium erythraea*. Their findings highlight a critical time window where shifts in cytokinin, auxin, and carbohydrate balance appear to drive regeneration, offering new insight into the physiological basis of *in vitro* morphogenesis. Ndathe and Kato addressed the role of phosphatidic acid (PA) in abscisic acid (ABA)-induced gene expression in *Arabidopsis thaliana* in association with stress responses. A specific subfamily of protein phosphatases 2C functions as ABA coreceptors, but this study shows that only a subset of this group is selectively inhibited by PA, emphasizing the importance of experimental validation when integrating signaling components into ABA response models. Tong et al. developed a novel method using ultra-high performance liquid chromatography–tandem mass spectrometry (UHPLC–MS/MS), combined with solid-phase extraction and chemical derivatization, to improve the detection of brassinosteroids (BRs) in plants. Applying this approach to *Brassica napus*, they quantified three BR types across different organs and revealed that BR levels are highest in flowers and decline with tissue maturation. Moreover, Zhang et al. provided a comprehensive overview of sulfated peptide hormones in plants, highlighting their roles in plant development, growth, and stress responses. The review details the production and modification of key peptide families, explores their signaling pathways and receptor interactions, and discusses the cross-talk between sulfated peptides and major phytohormones.

This Research Topic emphasizes the significance of the interactions among phytohormones and associated metabolites, including PA and sulfated peptides, as well as the importance of technical advances in measuring these compounds. Future research along these lines will deepen our understanding of how phytohormones function in dynamic environmental contexts.
